# *Hovenia dulcis* Suppresses the Growth of Huh7-Derived Liver Cancer Stem Cells by Inducing Necroptosis and Apoptosis and Blocking c-MET Signaling

**DOI:** 10.3390/cells13010022

**Published:** 2023-12-21

**Authors:** Mikyoung Kwon, Hye Jin Jung

**Affiliations:** 1 Department of Life Science and Biochemical Engineering, Graduate School, Sun Moon University, Asan 31460, Republic of Korea; alal5544@sunmoon.ac.kr; 2 Department of Pharmaceutical Engineering and Biotechnology, Sun Moon University, Asan 31460, Republic of Korea; 3 Genome-Based BioIT Convergence Institute, Sun Moon University, Asan 31460, Republic of Korea

**Keywords:** hepatocellular carcinoma, liver cancer stem cell, *Hovenia dulcis* Thunberg, necroptosis, apoptosis, c-MET, stemness marker

## Abstract

Liver cancer stem cells (LCSCs) contribute to the initiation, metastasis, treatment resistance, and recurrence of hepatocellular carcinoma (HCC). Therefore, exploring potential anticancer agents targeting LCSCs may offer new therapeutic options to overcome HCC treatment failure. *Hovenia dulcis* Thunberg (HDT), a tree from the buckthorn family found in Asia, exhibits various biological activities, including antifatigue, antidiabetic, neuroprotective, hepatoprotective, and antitumor activities. However, the therapeutic effect of HDT in eliminating LCSCs remains to be confirmed. In this study, we evaluated the inhibitory activity of ethanol, chloroform, and ethyl acetate extracts from HDT branches on the growth of Huh7-derived LCSCs. The ethyl acetate extract of HDT (EAHDT) exhibited the most potent inhibitory activity against the growth of Huh7 LCSCs among the three HDT extracts. EAHDT suppressed the in vitro self-renewal ability of Huh7 LCSCs and reduced tumor growth in vivo using the Huh7 LCSC-transplanted chick embryo chorioallantoic membrane model. Furthermore, EAHDT not only arrested the cell cycle in the G0/G1 phase but also induced receptor-interacting protein kinase 3/mixed-lineage kinase domain-like protein-mediated necroptosis and caspase-dependent apoptosis in Huh7 LCSCs in a concentration-dependent manner. Furthermore, the growth inhibitory effect of EAHDT on Huh7 LCSCs was associated with the downregulation of c-MET-mediated downstream signaling pathways and key cancer stemness markers. Based on these findings, we propose that EAHDT can be used as a new natural drug candidate to prevent and treat HCC by eradicating LCSCs.

## 1. Introduction

Hepatocellular carcinoma (HCC) accounts for approximately 90% of all liver cancer cases and is one of the leading causes of cancer-related deaths worldwide [1]. Key risk factors associated with HCC include liver cirrhosis, chronic hepatitis B or C viral infections, prolonged consumption of aflatoxin-contaminated foods, excessive alcohol intake, obesity, type 2 diabetes, and smoking [2]. While early-stage HCC can be addressed through surgical resection, advanced HCC requires systemic therapies, both targeted and non-targeted, such as atezolizumab, bevacizumab, sorafenib, and lenvatinib [3]. However, the recurrence rate, within 5 years after treatment, can go up to 70% [3]. Liver cancer stem cells (LCSCs) are recognized as major contributors to unfavorable clinical outcomes [4]. Despite their relatively small presence (1–3%) within tumor tissues, LCSCs play a crucial role in initiating, facilitating growth, fostering metastasis, resisting chemotherapy, and fueling recurrence in HCC. This is largely attributed to their capability for self-renewal, differentiation, and evading mechanisms of cell death [4]. The development and maintenance of LCSCs stem from the overexpression of distinct CSC markers, such as CD133, Sox2, Oct4, Nanog, aldehyde dehydrogenase 1A1 (ALDH1A1), and integrin α6, as well as the dysregulated activation of pivotal CSC-related signaling pathways, including Wnt/β-catenin, transforming growth factor-β, signal transducer and activator of transcription 3 (STAT3), and mesenchymal–epithelial transition factor (c-MET) [4,5,6,7,8,9]. Therefore, the downregulation of these markers and signaling molecules presents a promising strategy for eliminating LCSCs and, consequently, enhancing the efficacy of HCC treatment.

Cell death can be categorized into apoptosis and non-apoptotic cell death [10]. Apoptosis represents the most well-characterized form of programmed cell death, recognized as a fundamental therapeutic approach to suppress cancer cell growth [11]. Apoptosis is characterized by cellular shrinkage, membrane blebbing, nuclear condensation, DNA fragmentation, accumulation of reactive oxygen species (ROS), and the activation of mitochondrial- and death receptor-mediated pathways mediated by the caspase family [11,12]. However, cancer stem cells (CSCs) exhibit resistance to apoptosis through the activation of various anti-apoptotic mechanisms [13]. Resistance of CSCs to apoptosis can be overcome by inducing alternative non-apoptotic cell death pathways. Necroptosis is a programmed form of necrotic cell death that is regulated by well-defined signaling networks [14]. It displays distinctive morphological features compared to apoptosis, including increased cell volume, organelle swelling, disruption of plasma membrane integrity, and the formation of vacuoles. Necroptosis is induced through the activation of receptor-interacting protein kinase 3 (RIPK3) and its substrate, mixed-lineage kinase domain-like protein (MLKL), by the inhibition of caspase-8 [15]. Recent studies have revealed that several anticancer drugs induce CSC death via necroptosis [16]. For example, the nickel(II)-dithiocarbamate phenanthroline complex triggers necroptosis in breast CSCs by promoting the phosphorylation and oligomerization of MLKL [17]. Additionally, a specific inhibitor of ALDH1A, 673A, induces calcium-dependent necroptosis in ovarian CSCs and enhances their sensitivity to chemotherapy [18]. Consequently, anticancer drugs that stimulate necroptotic cell death may prove effective in eradicating LCSCs that exhibit resistance to apoptosis.

Plants have long been used as a rich source of anticancer drugs [19], and accumulating evidence has demonstrated that various plant-derived extracts and compounds possess anti-CSC activity [20,21,22,23,24]. Notably, the chloroform fraction of the methanol extract from *Sparassis crispa* effectively inhibits the proliferation, tumorsphere formation, and migration of cervical CSCs. This inhibition is achieved by promoting apoptosis and inhibiting the expression of cancer stemness markers [20]. The chloroform extract of *Citrus unshiu* Markovich peel, along with its active constituents, hesperidin and hesperetin, demonstrates the suppression of cancer stem-like properties in both cervical cancer and glioblastoma cells [21]. The chloroform extract of *Portulaca oleracea* exerts potent cytotoxic activity against glioblastoma stem cells, inducing apoptosis and downregulating crucial stemness regulatory factors including Sox2, Oct4, and STAT3 [22]. The flavonoid apigenin inhibits the self-renewal and invasion abilities of glioblastoma stem cells through the suppression of c-MET signaling [23]. Stilbenes, represented by resveratrol and pterostilbene, suppress the growth and migration of cervical CSCs by downregulating specific stem cell markers and STAT3 signaling [24]. Thus, plant-derived bioactive compounds represent valuable natural medicines for targeting CSCs.

*Hovenia dulcis* Thunberg (HDT), a tree indigenous to the buckthorn family in Asia, is renowned for its diverse biological activities, including antifatigue, antidiabetic, neuroprotective, hepatoprotective, antiangiogenic, and antitumor properties [25]. HDT is rich in various bioactive compounds such as flavonoids, terpenoids, alkaloids, polysaccharides, and organic acids [25]. Previous studies have revealed the significant cytotoxic effects of HDT extracts on numerous cancer cell lines, including breast, lung, cervical, colon, and liver cancer cells [26]. Additionally, the ethanol extract of HDT branches, along with its principal active component, ampelopsin, has been shown to effectively suppress tumor angiogenesis by inhibiting the expression of hypoxia-inducible factor-1α and vascular endothelial growth factor in HCC cells [27]. Nonetheless, the anti-CSC potential of HDT has remained unexplored. In this study, we assess the inhibitory capabilities of ethanol, chloroform, and ethyl acetate extracts derived from HDT branches on the growth of Huh7-derived LCSCs. Furthermore, we elucidate the mechanisms underlying the induction of necroptosis and apoptosis, as well as the downregulation of stemness-related factors, by the ethyl acetate extract of HDT (EAHDT), which exhibited the most potent growth-inhibiting effect in Huh7 LCSCs.

## 2. Materials and Methods

### 2.1. Materials

Dried HDT was collected from Byeonggok-myeon, Hamyang-gun, Gyeongsangnam-do, South Korea and purchased from Cheongundang Nongsan (Gyeongnam, South Korea). The original specimen of HDT (specimen number NCB-HDT-2022) was deposited at the Genome-based BioIT Convergence Institute of Sun Moon University (Asan, South Korea). Chloroform (99.9%), ethyl alcohol (99.9%), ethyl acetate (99.8%), acetonitrile (99.9%), and sodium carbonate anhydrous were purchased from Daejung (Daejeon, South Korea). Trifluoroacetic acid (TFA, 99.0%) was purchased from Samchun (Pyeongtaek, South Korea). Gallic acid, Folin–Denis’ reagent, hematoxylin, eosin, heparin, extracellular matrix (ECM) gel from Engelbreth-Holm-Swarm murine sarcoma, 4′,6-diamidine-2′-phenylindole dihydrochloride (DAPI), and dichlorodihydrofluorescein diacetate (DCFH-DA) were purchased from Sigma-Aldrich (St. Louis, MO, USA). Dulbecco’s Modified Eagle’s Medium/Nutrient Mixture F-12 (DMEM/F-12) and DMEM media were purchased from HyClone (Marlborough, MA, USA), and epidermal growth factor (EGF) and basic fibroblast growth factor (bFGF) were purchased from Prospecbio (East Brunswick, NJ, USA). Fetal bovine serum (FBS), B-27 serum free supplement, L-glutamine, penicillin/streptomycin, and trypsin were purchased from Gibco (Grand Island, NY, USA). Penicillin-streptomycin-amphotericin B and Accutase were purchased from Lonza (Walkersville, MD, USA). Necrostatin-1 (#ab141053) and anti-β-actin antibody (#ab6276) were purchased from Abcam (Cambridge, UK). Antibodies against cleaved caspase-9 (#9501), cleaved caspase-3 (#9661), cleaved caspase-8 (#9748), PARP (#9542), integrin α6 (#3750), CD133 (#64326), ALDH1A1 (#12035), Nanog (#3580), Oct4 (#2750), Sox2 (#3579), phospho-c-MET (#3077), phospho-ERK1/2 (#9101), ERK1/2 (#9102), phospho-AKT (#4060), AKT (#9272), phospho-NF-κB (#3033), NF-κB (#8242), rabbit IgG (#7074), and mouse IgG (#7076) were purchased from Cell Signaling Technology (Danvers, MA, USA). Antibodies against c-MET (#A0040), phospho-RIPK3 (#AP1260), and phospho-MLKL (#AP1244) were purchased from ABclonal (Woburn, MA, USA).

### 2.2. Extraction of HDT

Crushed HDT (50 g) was added to ethanol, ethyl acetate, and chloroform (350 mL), stirred at room temperature for 72 h, and then filtered using gauze and a 0.22 µm syringe filter. Each extract was concentrated using a rotary evaporator and then lyophilized. The yields of ethanol, ethyl acetate, and chloroform extracts from HDT were 0.6254% (625.4 mg), 0.3742% (374.2 mg), and 1.461% (1461 mg), respectively. Each HDT extract was dissolved in dimethyl sulfoxide (DMSO) at a concentration of 100 mg/mL and used in experiments. The negative control groups were treated with equal volumes of DMSO.

### 2.3. Total Polyphenol Analysis

The Folin–Ciocalteu method was used to assess the polyphenol content of HDT extracts [28]. Each HDT extract was prepared at concentrations of 60, 80, and 100 μg/mL, and 150 µL of the extract was mixed with 300 µL of 2 N Folin–Ciocalteu reagent. Then, the mixture was added to 1050 µL of 700 mM Na₂CO₃ and incubated in the dark for 30 min. Absorbance was measured at 600 nm using a microplate reader (BioTek, Inc., Winooski, VT, USA). To quantify total polyphenols, a standard curve was obtained using gallic acid, and the standard curve equation was y = 0.0841x + 0.0309 (R² = 0.9926). Total polyphenol content was expressed as mg GAE/mL.

### 2.4. HPLC Analysis of EAHDT

High-performance liquid chromatography (HPLC) analysis of EAHDT, ampelopsin, and quercetin was performed using an UltiMate™ 3000 UHPLC (Thermo Fisher Scientific, Waltham, MA, USA) and a Shim-pack GIS C18 column (5 μm, 4.6 mm × 250 mm, Shimadzu Co., Kyoto, Japan). The binary mobile phase consisted of solvent A (water with 0.1% TFA) and solvent B (100% acetonitrile). The mobile phase flow rate and oven temperature were maintained at 1.0 mL/min and 50 °C, respectively. The linear gradient elution conditions were set at 5% B to 90% B (0–28 min), 90% B (28–30 min), 90% B to 10% B (30–33 min), and 10% B (33–35 min). Chromatograms were obtained at a wavelength of 270 nm.

### 2.5. LC-MS Analysis of EAHDT

Liquid chromatography–mass spectrometry (LC-MS) analysis of EAHDT was performed using an ACQUITY UPLC/SYNAPT G2-S QTOF (Waters, Milford, MA, USA) and an ACQUITY UPLC^®^BEH C18 column (1.7 μm, 2.1 mm × 100 mm, Waters, Milford, MA, USA). The binary mobile phase consisted of solvent A (water with 0.1% TFA) and solvent B (acetonitrile with 0.1% TFA). The mobile phase flow rate and oven temperature were maintained at 0.3 mL/min and 35 °C, respectively. The elution conditions were set at 95% A and 5% B for 0–7 min, 0% A and 100% B for 7–9.5 min, and 95% A and 5% B for 9.5–12 min. Then, the sample was vaporized at 120 °C, dissolved at 300 °C, injected into the device at a pressure of 600 L/h and 6.0 bar, and analyzed with a capillary voltage of 2.5 kV. The results obtained were analyzed using the UNIFI (Waters, Milford, MA, USA) program.

### 2.6. Cell Culture

Huh7 human hepatocellular carcinoma cell line was purchased from Korean Cell Line Bank (Seoul, South Korea). Adherent cells were grown in DMEM containing 10% FBS and 1% penicillin-streptomycin-amphotericin B and subcultured using trypsin. Tumorsphere cells with stem-like properties were propagated in serum-free DMEM/F-12 containing 1 × B-27, 5 µg/mL heparin, 2 mM L-glutamine, 20 ng/mL bFGF, 20 ng/mL EGF, and 1% penicillin/streptomycin and subcultured using Accutase [29]. All cells were maintained at 37 °C in a humidified 5% carbon dioxide incubator (Thermo Scientific, Vantaa, Finland).

### 2.7. Extreme Limiting Dilution Analysis (ELDA)

The self-renewal capacity of cells was determined using in vitro ELDA [29]. Single cells were added to 96-well culture plates at different cell numbers per well (5–500 cells/well) and incubated for 72 h. The presence and number of tumorspheres (>50 µm) in each well were observed and quantified using an optical microscope (Olympus, Tokyo, Japan). Stem cell frequencies were analyzed using ELDA software (http://bioinf.wehi.edu.au/software/elda/) (accessed on 13 April 2023).

### 2.8. Measurement of Cell Proliferation

Cell proliferation was measured using the CellTiter-Glo^®^ luminescence assay (Promega, Madison, WI, USA) [29]. Huh7-derived LCSCs were added to 96-white well culture plates at 3 × 10³ cells per well and exposed to different concentrations (0–500 µg/mL) of HDT extracts. Following incubation for 7 days, 20 µL of substrate solution was added to each well, and luminescence was measured using a microplate reader (BioTek, Inc., Winooski, VT, USA). IC₅₀ values were analyzed with the curve-fitting program in GraphPad Prism 6 (GraphPad Software, La Jolla, CA, USA).

### 2.9. Analysis of Tumorsphere Formation

Huh7-derived LCSCs were added to 96-well culture plates at 3 × 10³ cells per well and exposed to different concentrations (0–62.5 µg/mL) of HDT extracts. Following incubation for 7 days, the formed tumorspheres (>70 µm) were observed and counted using an optical microscope (Olympus, Tokyo, Japan).

### 2.10. Cell Cycle Analysis

Huh7-derived LCSCs were added to 60 mm culture dishes at 2 × 10⁵ cells per well and exposed to 5, 10, 20, and 40 µg/mL of EAHDT. Following incubation for 48 h, cells were harvested, fixed with 200 µL of 70% ethanol for at least 3 h at −20 °C, and then stained with 200 µL of Muse^®^ Cell Cycle reagent (Luminex, Austin, TX, USA) for 30 min [30]. Cell-cycle distribution of stained cells was analyzed using the Guava^®^ Muse^®^ Cell Analyzer (MuseSoft_V1.8.0.3; Luminex, Austin, TX, USA).

### 2.11. Cell Death Analysis

Huh7-derived LCSCs were added to 60 mm culture dishes at 2 × 10⁵ cells per well and exposed to 5, 10, 20, and 40 µg/mL of EAHDT. Following incubation for 48 h, cells were harvested and stained with 100 µL of Muse^®^ Annexin V & Dead Cell reagent (Luminex, Austin, TX, USA) for 20 min [29,30]. Cell death was analyzed using the Guava^®^ Muse^®^ Cell Analyzer (MuseSoft_V1.8.0.3; Luminex, Austin, TX, USA).

### 2.12. Hematoxylin & Eosin (H&E) Staining

Huh7-derived LCSCs were added to 24-well culture plates at 5 × 10^4^ cells per well and exposed to 10, 20, and 40 µg/mL of EAHDT. Following incubation for 24 h, cells were fixed with 4% formaldehyde solution at room temperature and then stained with H&E for 7 min. Stained cells were observed under an optical microscope (Olympus, Tokyo, Japan). Doxorubicin (2 µM) was used as a control drug for apoptosis. Necroptotic and apoptotic cells were counted and further quantified.

### 2.13. Nuclear Staining with DAPI

Huh7-derived LCSCs were added to 24-well culture plates at 1 × 10^5^ cells per well and exposed to 5, 10, 20, and 40 µg/mL of EAHDT. Following incubation for 24 h, cells were harvested and stained with 20 µg/mL of DAPI for 1 h in a cell incubator (37 °C, 5% CO₂). The nuclear morphology of cells was observed using a fluorescence microscope (Optinity KI-2000F, Korea Lab Tech, Seung Nam, Korea) [30]. Chromatin-cleaved cells were counted and further quantified.

### 2.14. ROS Measurement with DCFH-DA

Huh7-derived LCSCs were added to 24-well culture plates at 1 × 10^5^ cells per well and exposed to 5, 10, 20, and 40 µg/mL of EAHDT. Following incubation for 24 h, cells were stained with 10 μM of DCFH-DA for 30 min in a cell incubator (37 °C, 5% CO₂). DCF fluorescence was observed using a fluorescence microscope (Optinity KI-2000F, Korea Lab Tech, Seongnam, Korea) [30]. The intensity of DCF fluorescence was measured by densitometry and further quantified.

### 2.15. Immunoblotting Analysis

Huh7-derived LCSCs were added to 100 mm culture dishes at 2 × 10^6^ cells per well and exposed to 10, 20, and 40 µg/mL of EAHDT. Following incubation for 48 h, cells were harvested and lysed with RIPA lysis buffer (ATTO, Tokyo, Japan). Whole-cell lysates were separated by 7.5–15% sodium do-decyl sulfate-polyacrylamide gel electrophoresis (SDS-PAGE), followed by electrotransfer to polyvinylidene fluoride (PVDF) membranes. Blots were blocked for 1 h in 5% nonfat milk in Tris-buffered saline containing 0.1% Tween-20 (TBST) and then incubated with primary antibodies (dilution 1:2000–1:10,000) overnight at 4 °C. After incubation with horseradish peroxidase-conjugated secondary antibodies (dilution 1:3000) for 1 h, immunolabeling was detected using an enhanced chemiluminescence kit (Bio-Rad Laboratories, Hercules, CA, USA). Band density was analyzed using ImageJ 1.5 software (NIH, Bethesda, MD, USA), and protein expression levels were quantified by calculating the band intensity of the target protein against β-actin [29,30].

### 2.16. Chick Embryo Chorioallantoic Membrane (CAM) Assay

Fertilized chicken eggs were incubated in an incubator at 37 °C for 7 days and then turned over to create a small window of less than 1 cm. Huh7-derived LCSCs (2 × 10^6^ cells/10 µL), EAHDT (100 µg), and ECM gel (10 mg/mL, 40 µL/egg) were mixed and solidified in a cell incubator (37 °C, 5% CO₂) for 1 h. The mixture was then injected onto the CAM surface. Following incubation for 7 days, the size and weight of the formed tumors were analyzed [29,30].

### 2.17. Statistical Analysis

Data were expressed as mean ± standard deviation (SD), and statistical analysis was performed using ANOVA with Tukey’s post hoc test (SPSS software version 9.0; Chicago, IL, USA) [29,30]. *p* < 0.05 was considered a statistically significant difference.

## 3. Results

### 3.1. Total Polyphenol and HPLC Analysis of Extracts from HDT

**Figure 1:**
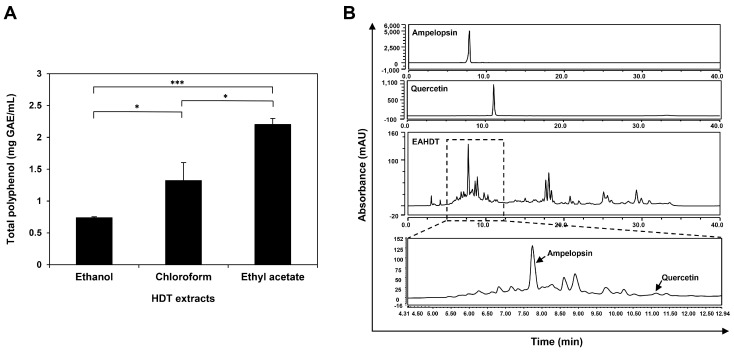
Total polyphenol content and HPLC analysis of HDT extracts. (**A**) Total polyphenol content of ethanol, chloroform, and ethyl acetate extracts from HDT. (**B**) HPLC analysis of EAHDT. * *p* < 0.05, *** *p* < 0.001.

Polyphenols are a class of chemicals found in plants known for their diverse biological activities in preventing and treating human diseases, including cancer [31]. Notably, HDT is rich in polyphenols such as ampelopsin and quercetin [25]. To assess the total polyphenol content of ethanol, chloroform, and ethyl acetate extracts obtained from HDT branches, we employed the Folin–Ciocalteu method [28]. As illustrated in Figure 1A, EAHDT exhibited a higher polyphenol content (2.20 mg GAE/mL) than the ethanol and chloroform extracts (0.74 and 1.32 mg GAE/mL, respectively). Subsequently, the presence and quantity of ampelopsin and quercetin in EAHDT were confirmed by comparing HPLC-ultraviolet chromatograms of standard samples and EAHDT under identical analytical conditions. Ampelopsin and quercetin were detected, with retention times of 7.74 and 11.15 min, respectively (Figure 1B). The estimated contents of ampelopsin and quercetin in EAHDT were found to be 0.813 μg/g and 0.193 μg/g, respectively. Furthermore, LC-MS analysis identified the presence of two compounds with molecular masses of 320.7401 and 303.0496 *m*/*z*, respectively, in EAHDT. Thus, these results affirmed that EAHDT possesses the highest polyphenol content among the three HDT extracts and contains trace amounts of ampelopsin and quercetin.

### 3.2. Propagation of Huh7-Derived LCSCs by Spheroid Cell Culture

**Figure 2:**
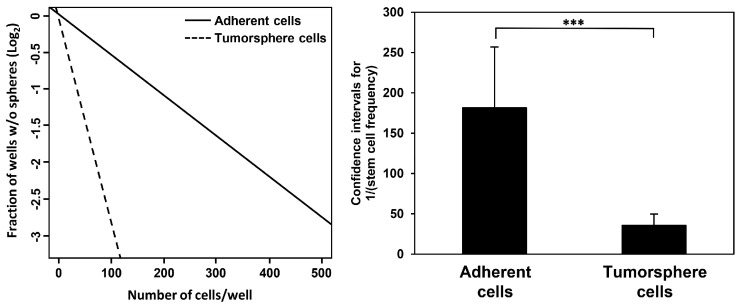
Comparison of the self-renewal capacity of Huh7 adherent and tumorsphere cells using extreme limiting dilution analysis (ELDA). Huh7 adherent and tumorsphere cells were seeded at exceedingly low cell numbers (5–500 cells) per well, incubated for 72 h, and the presence and quantity of tumorspheres in each well were quantified. Stem cell frequencies were analyzed using ELDA software (http://bioinf.wehi.edu.au/software/elda/) (accessed on 13 April 2023). *** *p* < 0.001.

In our previous study, we established that a three-dimensional spheroid cell culture employing serum-free medium supplemented with EGF and bFGF efficiently propagates LCSC populations derived from HCC cell lines [29]. Notably, Huh7 tumorsphere cells cultivated in serum-free conditions displayed significantly elevated levels of key stemness markers in comparison to Huh7 adherent cells grown in serum-containing medium [32]. To further confirm the stem cell properties of Huh7 tumorsphere cells, we conducted an extreme limiting dilution analysis (ELDA). ELDA is a well-established method for quantifying the capacity of individual stem cells to divide, proliferate, and form tumorspheres [33]. Huh7 adherent and tumorsphere cells were seeded at exceedingly low cell numbers (5–500 cells) per well, incubated for 72 h, and the presence and quantity of tumorspheres in each well were quantified. Stem cell frequencies were analyzed using ELDA software (http://bioinf.wehi.edu.au/software/elda/) (accessed on 13 April 2023). The results indicated that Huh7 tumorsphere cells exhibited a five-fold higher frequency of stem cells in comparison to Huh7 adherent cells, indicating the exceptional self-renewal ability characteristic of stem cells (Figure 2). Consequently, we expanded Huh7-derived LCSCs via tumorsphere cell culture and used them for all experiments in this study.

### 3.3. HDT Extracts Inhibit Proliferation and Tumorsphere Formation of Huh7-Derived LCSCs

**Figure 3:**
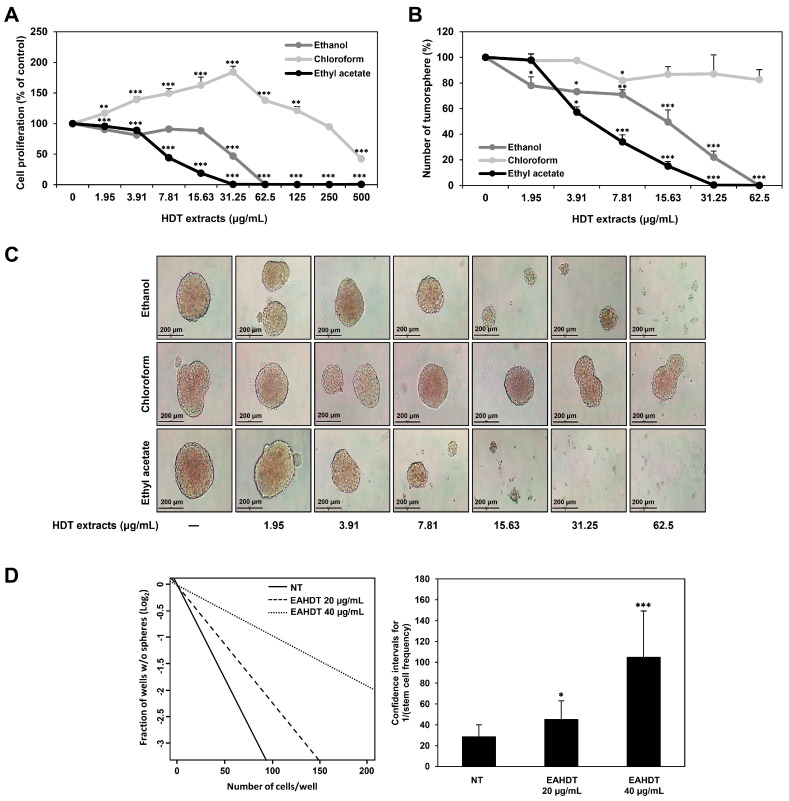
Effect of HDT extracts on proliferation and tumorsphere formation of Huh7-derived LCSCs. (**A**) Effect of HDT extracts on the proliferation of Huh7 LCSCs. Cells were exposed to different concentrations (0–500 µg/mL) of HDT extracts for 7 days, and then cell proliferation was analyzed using the CellTiter-Glo^®^ luminescence assay. (**B**,**C**) Effect of HDT extracts on the tumorsphere-forming ability of Huh7 LCSCs. After the cells were exposed to different concentrations (0–62.5 µg/mL) of HDT extracts for 7 days, the formed tumorspheres were observed and counted. (**D**) Effect of EAHDT on the self-renewal ability of Huh7 LCSCs using ELDA. Cells were exposed to EAHDT (20, 40 µg/mL) for 4 days, and stem cell frequencies were analyzed using ELDA software (http://bioinf.wehi.edu.au/software/elda/) (accessed on 13 April 2023). * *p* < 0.05, ** *p* < 0.01, *** *p* < 0.001 vs. the control.

To evaluate the inhibitory effects of ethanol, chloroform, and ethyl acetate extracts of HDT on LCSC proliferation, we employed a luminescent ATP detection assay. Huh7-derived LCSCs were treated with various concentrations (0–500 µg/mL) of HDT extracts for 7 days. The results revealed that EAHDT exhibited the most potent inhibitory effect on Huh7 LCSC proliferation compared to the other two HDT extracts (Figure 3A). The IC₅₀ values for the ethanol, chloroform, and ethyl acetate extracts of HDT were measured at 33.0, 301.0, and 7.5 µg/mL, respectively. Next, the tumorsphere formation ability of Huh7 LCSCs was assessed after treatment with each extract (0–62.5 µg/mL) for 7 days. EAHDT was found to significantly reduce the number and size of tumorspheres compared to the ethanol and chloroform extracts (Figure 3B,C). Collectively, EAHDT displayed the most robust inhibitory activity against Huh7 LCSC growth among the three HDT extracts. Based on these findings, we further investigated the effect of EAHDT on Huh7 LCSCs.

To determine the effect of EAHDT on the self-renewal capacity of Huh7 LCSCs, we performed ELDA. Treating Huh7 LCSCs with EAHDT for 4 days led to a concentration-dependent decrease in the frequency of stem cells compared to the untreated control (Figure 3D), demonstrating the effectiveness of EAHDT in suppressing the self-renewal of Huh7 LCSCs.

### 3.4. EAHDT Induces Cell-Cycle Arrest, Necroptosis, and Apoptosis in Huh7-Derived LCSCs

**Figure 4:**
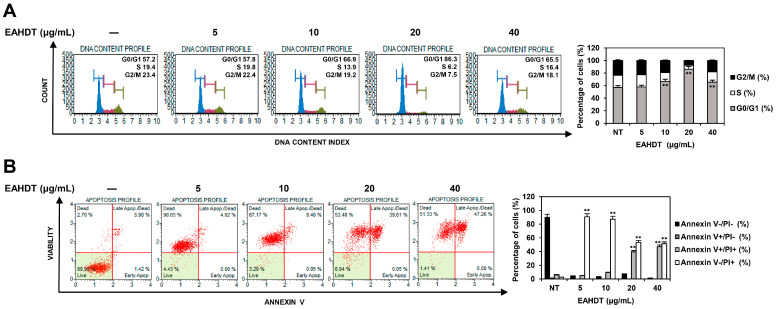
Effect of EAHDT on cell cycle and cell death of Huh7-derived LCSCs. (**A**,**B**) Huh7 LCSCs were exposed to EAHDT (5, 10, 20, 40 μg/mL) for 48 h. Cell-cycle distribution (**A**) and cell death (**B**) were analyzed using the Guava^®^ Muse^®^ Cell Analyzer. ** *p* < 0.01 vs. the control.

Dysregulated cell-cycle progression is implicated in endless cancer cell proliferation, metastasis, and recurrence [34]. To determine whether EAHDT inhibits the proliferation of Huh7-derived LCSCs by affecting cell-cycle progression, Huh7 LCSCs were exposed to EAHDT at concentrations of 5, 10, 20, and 40 µg/mL for 48 h. Subsequently, cell-cycle distribution was analyzed by flow cytometry using Muse^®^ Cell Cycle reagent. As demonstrated in Figure 4A, treatment with 5, 10, and 20 µg/mL concentrations of EAHDT increased the cell population in the G0/G1 phase to 57.8%, 66.9%, and 86.3%, respectively, in contrast to the untreated control (57.2%). These results indicate that EAHDT inhibits Huh7 LCSC proliferation by arresting the cell cycle in the G0/G1 phase. However, at 40 µg/mL of EAHDT, cell-cycle arrest in the G0/G1 phase was reduced to 65.5% compared to 20 µg/mL treatment, suggesting that high doses of EAHDT may promote strong cell death rather than inhibiting cell proliferation.

The induction of programmed cell death, such as apoptosis and necroptosis, is a key therapeutic strategy to suppress the growth of CSCs [35]. To investigate whether the growth inhibitory effect of EAHDT in Huh7-derived LCSCs was associated with the induction of programmed cell death, Huh7 LCSCs were exposed to 5, 10, 20, and 40 µg/mL of EAHDT for 48 h. Subsequently, cells were stained with Muse^®^ Annexin V & Dead Cell reagent and analyzed using flow cytometry. As depicted in Figure 4B, treatment with 5 and 10 µg/mL concentrations of EAHDT remarkably increased the percentage of necroptotic cells to 90.65% and 87.17%, respectively, compared to the untreated control (2.70%). However, treatment with 20 and 40 µg/mL of EAHDT decreased the percentage of necroptotic cells to 53.40% and 51.33% and increased the percentage of late apoptotic cells to 39.61% and 47.26%, respectively. These findings suggest that EAHDT inhibits the growth of Huh7 LCSCs by inducing both necroptosis and apoptosis.

### 3.5. EAHDT Induces Features of Both Necroptosis and Apoptosis in Huh7-Derived LCSCs

**Figure 5:**
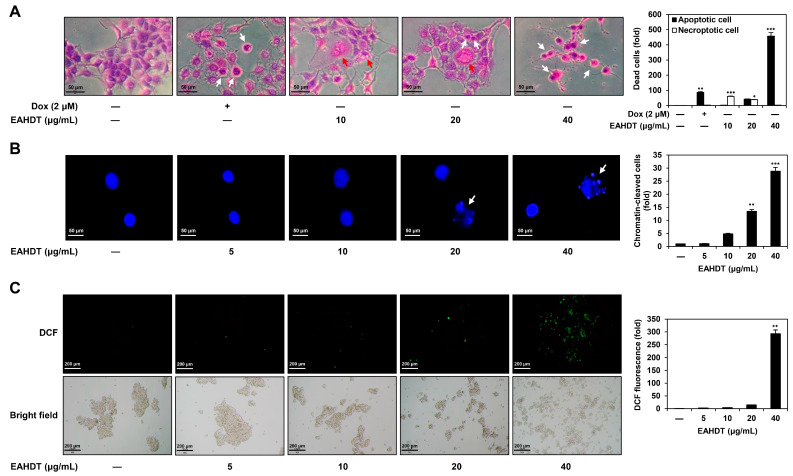
EAHDT induces features of both necroptosis and apoptosis in Huh7-derived LCSCs. (**A**–**C**) Huh7 LCSCs were exposed to EAHDT (5, 10, 20, 40 μg/mL) for 24 h. (**A**) Cell morphology was observed by staining with H&E. Doxorubicin (Dox) is a control drug that induces apoptosis. Necroptotic and apoptotic cell morphologies are indicated by red and white arrows, respectively. (**B**) Changes in nuclear morphology were observed by staining with DAPI under a fluorescence microscope. Chromatin cleavage and condensation are indicated by white arrows. (**C**) ROS levels were detected by DCF fluorescence. * *p* < 0.05, ** *p* < 0.01, *** *p* < 0.001 vs. the control.

We further investigated the necroptotic and apoptotic effects of EAHDT in Huh7-derived LCSCs. Following treatment with 10, 20, and 40 µg/mL of EAHDT for 24 h, we observed changes in cell morphology by conducting H&E staining. As shown in Figure 5A, doxorubicin, a control drug for apoptosis, induced cell shrinkage and membrane blebbing, which are typical morphological characteristics of apoptosis. In contrast, EAHDT treatment resulted in both necroptotic and apoptotic cell morphology. At a concentration of 10 µg/mL, EAHDT exhibited morphological features of necroptosis, including cell swelling and vacuole formation. At a concentration of 20 µg/mL, it induced both necroptotic and apoptotic cell morphology, and at 40 µg/mL, it caused apoptotic cell morphology (Figure 5A).

Necroptotic cells typically maintain the integrity of their nucleus, whereas apoptotic cells undergo nuclear condensation and fragmentation [11,15]. To elucidate the effect of EAHDT on necroptosis or apoptosis, depending on the concentration, Huh7 LCSCs were treated with EAHDT at 5, 10, 20, and 40 µg/mL for 24 h, and changes in nuclear morphology were assessed through DAPI staining. As shown in Figure 5B, chromatin structure remained unaltered upon treatment with 5 and 10 µg/mL of EAHDT. However, EAHDT at 20 and 40 µg/mL resulted in chromatin cleavage and condensation. These observations indicate that EAHDT induces necroptosis at lower doses and predominantly promotes apoptosis at higher doses in Huh7 LCSCs.

Excessive ROS production plays an important role in the activation of apoptosis through both intrinsic and extrinsic pathways [36]. To investigate the influence of EAHDT on ROS production in Huh7 LCSCs, we measured intracellular ROS levels using DCFH-DA following 24 h of treatment with 5, 10, 20, and 40 µg/mL of EAHDT. As depicted in Figure 5C, EAHDT at 40 µg/mL significantly increased ROS production compared to the untreated control, indicating that higher doses of EAHDT are associated with increased apoptotic cell death. In summary, these findings suggest that EAHDT induces both necroptosis and apoptosis in Huh7-derived LCSCs.

### 3.6. Dose-Dependent Activation of Necroptotic and Apoptotic Pathways by EAHDT in Huh7-Derived LCSCs

**Figure 6:**
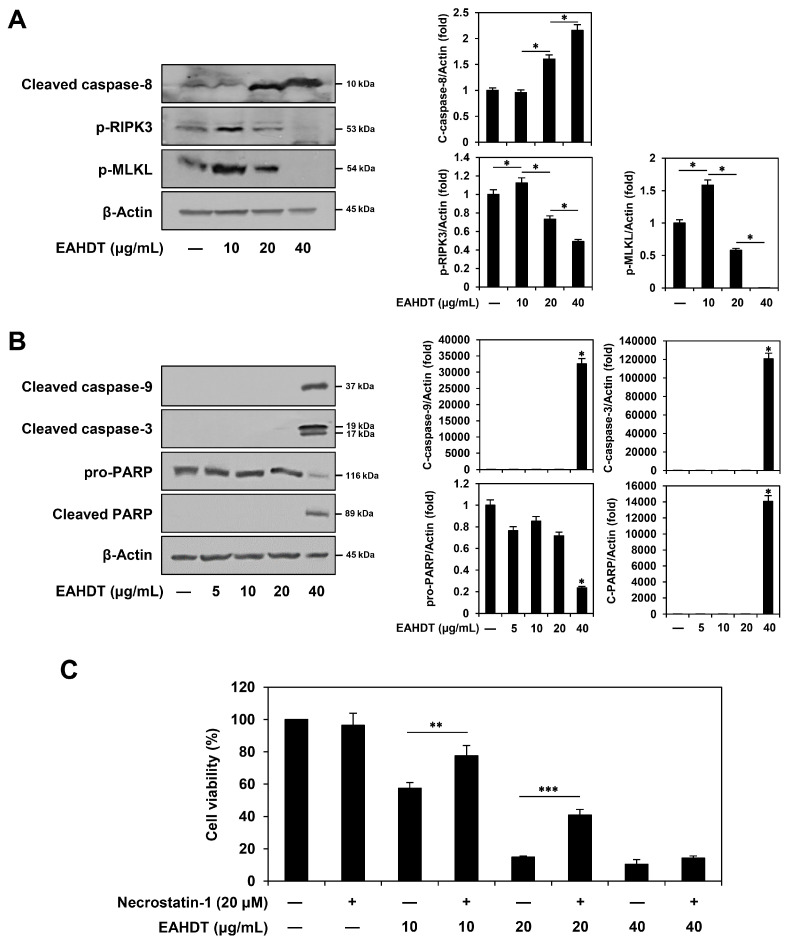
Dose-dependent activation of necroptotic and apoptotic pathways by EAHDT in Huh7-derived LCSCs. (**A**,**B**) Huh7 LCSCs were exposed to EAHDT (5, 10, 20, 40 μg/mL) for 48 h. Protein levels of necroptosis and apoptosis regulators were detected by Western blot analysis using specific antibodies and further quantified by densitometry. β-Actin levels were used as a loading control. (**C**) Huh7 LCSCs were pretreated with necrostatin-1 (20 μM) for 1 h and then exposed to EAHDT (10, 20, 40 μg/mL) for 48 h. Cell viability was analyzed using the CellTiter-Glo^®^ luminescence assay. * *p* < 0.05, ** *p* < 0.01, *** *p* < 0.001.

We next examined the effects of EAHDT on the expression of molecular markers that play a critical role in the activation of necroptosis and apoptosis in Huh7-derived LCSCs. When caspase-8, the initiator of the extrinsic apoptotic pathway, is inhibited, RIPK3 and its substrate MLKL become activated through phosphorylation, leading to necroptosis [15]. As shown in Figure 6A, treatment with 10 µg/mL of EAHDT led to caspase-8 inactivation and an increase in phosphorylated RIPK3 and MLKL levels in Huh7 LCSCs. However, as EAHDT concentrations increased to 20 and 40 µg/mL, a dose-dependent shift occurred in the mode of cell-death induction, with elevated levels of cleaved caspase-8 and reduced expression of phosphorylated RIPK3 and MLKL. These results indicate that EAHDT shifts the mode of cell-death induction from necroptosis to apoptosis as the treatment concentration increases. This observation was consistent with the increased levels of cleaved caspase-9, caspase-3, and poly(ADP)-ribose polymerase (PARP), key regulators of the intrinsic apoptotic pathway [12], which were significantly upregulated only at the higher EAHDT concentration of 40 µg/mL (Figure 6B). In addition, we confirmed the necroptotic effect of EAHDT using necrostatin-1, a selective necroptosis inhibitor. Figure 6C shows that necrostatin-1 partially rescued the viability of Huh7 LCSCs that had been inhibited by EAHDT. Notably, the restorative effect of necrostatin-1 was higher with 10 and 20 µg/mL EAHDT treatments than with the 40 µg/mL treatment. These findings collectively suggest that, at lower doses, EAHDT predominantly activates the necroptotic pathway rather than the apoptotic pathway in Huh7-derived LCSCs.

### 3.7. EAHDT Downregulates Stem Cell Markers and c-MET Signaling in Huh7-Derived LCSCs

**Figure 7:**
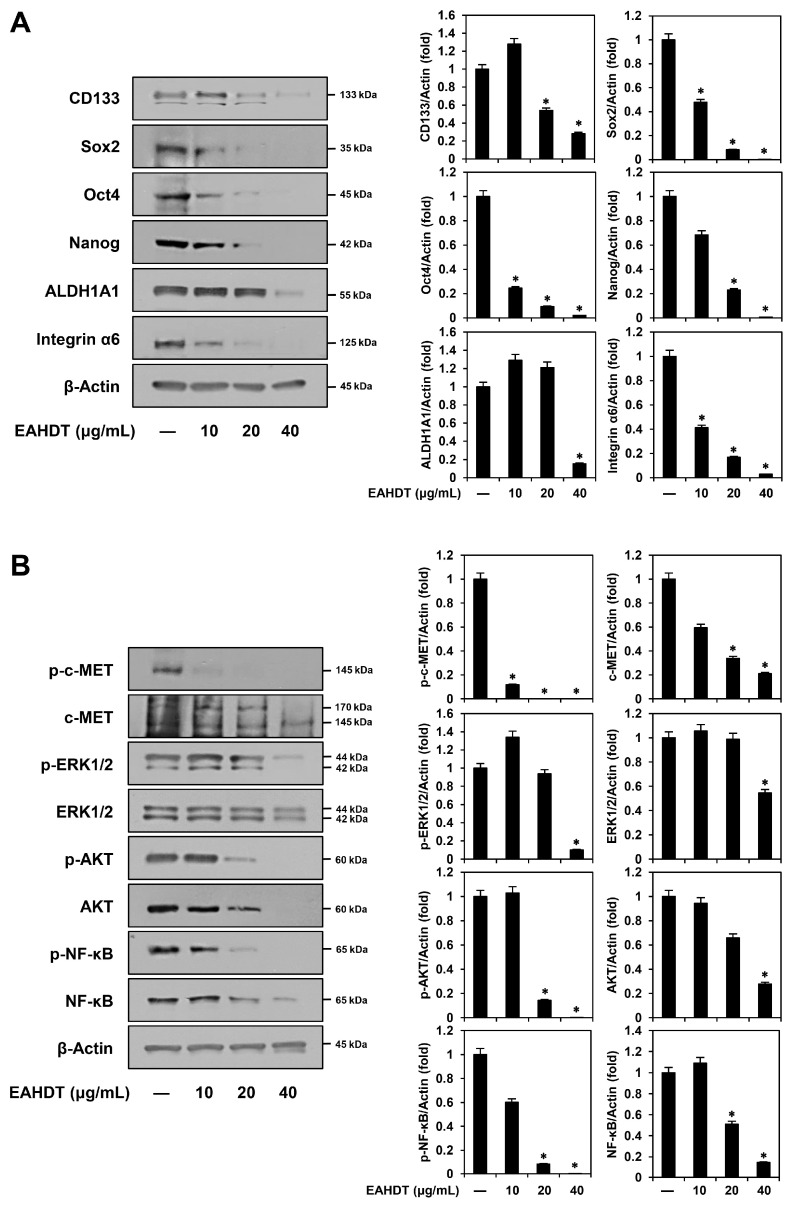
Effect of EAHDT on stemness markers and c-MET-mediated signaling pathway in Huh7-derived LCSCs. (**A**,**B**) Huh7 LCSCs were exposed to EAHDT (10, 20, 40 μg/mL) for 48 h. Protein levels were detected by Western blot analysis using specific antibodies and further quantified by densitometry. β-Actin levels were used as a loading control. * *p* < 0.05 vs. the control.

Abnormal expression and activation of stem cell-related markers and signaling pathways contribute to the aggressive and malignant characteristics of LCSCs [4,5]. Therefore, inhibiting these specific stem cell markers and signaling molecules has been recognized as an effective strategy to eradicate LCSCs [4,5]. We evaluated the effect of EAHDT on the expression of key stemness markers in Huh7-derived LCSCs. As shown in Figure 7A, EAHDT treatment effectively reduced the expression levels of cell surface markers, including CD133 and integrin α6, intracellular transcription factors, such as Sox2, Oct4, and Nanog, and the cytoplasmic detoxification enzyme ALDH1A1. These data highlight the association between the growth-inhibitory effect of EAHDT on Huh7 LCSCs and the downregulation of these CSC markers.

Activated c-MET plays a pivotal role in promoting liver cancer stemness by upregulating downstream signaling molecules, including extracellular signal-regulated kinase 1/2 (ERK1/2), protein kinase B (AKT), and nuclear factor kappa B (NF-κB) [37]. Therefore, inhibiting these c-MET-mediated signaling pathways may hinder the growth and maintenance of LCSCs. Figure 7B shows that EAHDT significantly inhibited both the phosphorylated and unphosphorylated forms of c-MET, ERK1/2, AKT, and NF-κB in Huh7-derived LCSCs. Notably, EAHDT exhibited a more pronounced inhibitory effect on the phosphorylated forms compared to the unphosphorylated forms. These results suggest that EAHDT may suppress the growth of Huh7 LCSCs by inactivating c-MET and its key downstream effectors, including ERK1/2, AKT, and NF-κB.

### 3.8. Inhibition of In Vivo Tumor Growth of Huh7-Derived LCSCs by EAHDT in a CAM Model

**Figure 8:**
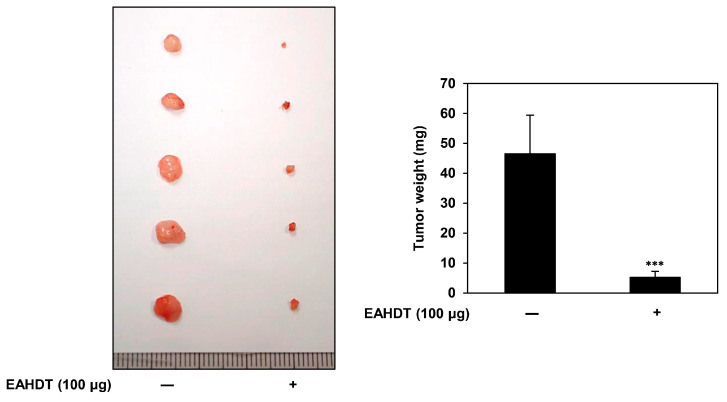
Inhibition of in vivo tumor growth of Huh7-derived LCSCs by EAHDT in a CAM model. The solidified ECM gel mixed with Huh7 LCSCs and 100 µg of EAHDT was injected onto the CAM surface and incubated for 7 days. The size and weight of the formed tumor were measured. *** *p* < 0.001 vs. the control.

To investigate the impact of EAHDT on the tumorigenic potential of Huh7-derived LCSCs in vivo, we employed the chick embryo chorioallantoic membrane (CAM) model. We injected a solidified ECM gel mixed with Huh7 LCSCs and 100 µg of EAHDT onto the CAM surface and incubated it for 7 days. Subsequently, we measured the size and weight of the formed tumor. The results revealed that the tumor weight in the control group was 46.5 mg, whereas the tumor weight in the EAHDT treatment group was 5.2 mg (Figure 8). These findings demonstrate the effective inhibition of the in vivo tumorigenic ability of Huh7 LCSCs by EAHDT.

## 4. Discussion

HCC is a highly aggressive tumor frequently diagnosed in the advanced stages of chronic liver disease [1,2,3]. Despite the availability of several targeted therapies and immunotherapies for HCC treatment, including sorafenib, regorafenib, bevacizumab, and atezolizumab, their effectiveness remains limited in overcoming drug resistance, metastasis, and cancer recurrence [3]. This challenge is largely attributed to LCSCs, which play a central role in therapy resistance, as they possess self-renewal, multi-lineage differentiation, and metastatic abilities [4]. Therefore, the development of novel chemotherapeutic agents targeting LCSCs is of utmost importance to enhance liver cancer treatment and reduce mortality rates. Natural products are a valuable resource for new drug candidates over the past few decades [38]. Phytochemicals, including flavonoids, stilbenes, terpenoids, and alkaloids, have been widely explored for their potential in cancer prevention and treatment [39]. More recent studies have revealed the ability of plant extracts or compounds to suppress the stem-like properties of cancer cells [40]. For instance, resveratrol has been shown to induce caspase-mediated apoptosis and inhibit key transcription factors involved in stem cell maintenance, including Nanog and Oct4, in pancreatic CSCs [41]. Curcumin increases the sensitivity of conventional chemotherapy in colon cancer by targeting colon CSCs [42]. Quercetin reduces the migration, invasion, and tumorsphere formation of prostate CSCs, whereas apigenin inhibits the growth and metastatic potential of glioblastoma stem cells by blocking c-MET signaling [23,43]. Furthermore, plant extracts containing various functional ingredients have also exhibited potential anti-CSC effects [20,21,22,23,24,44]. Chloroform extracts from *Sparassis crispa, Citrus unshiu* Markovich, and *Portulaca oleracea* have been found to inhibit the self-renewal capabilities of cervical CSCs or glioblastoma stem cells through apoptosis induction and the suppression of stemness markers [20,21,22]. Therefore, plant extracts hold great potential as natural medicines for CSC eradication. In this study, for the first time, we evaluated the growth-inhibitory activity of HDT extract against LCSCs. Our results revealed that EAHDT exhibits stronger inhibitory effects on the growth of Huh7-derived LCSCs in comparison to ethanol and chloroform extracts. Furthermore, EAHDT not only effectively suppresses the in vitro self-renewal abilities of Huh7 LCSCs but also significantly reduces in vivo tumor growth in the Huh7 LCSC-transplanted CAM model. Additionally, the growth-inhibitory effect of EAHDT on LCSCs was also demonstrated in other HCC cells. EAHDT strongly inhibited the proliferation (IC_50_: 5.1 µg/mL) and tumorsphere-formation ability of Hep3B-derived LCSCs (Supplementary Materials, Figure S1). These results demonstrate the potential of EAHDT as a novel natural medicine for preventing and treating the stem-like properties of HCC cells.

Inducing apoptosis has been a powerful method in anticancer treatments [11]. However, CSCs often resist apoptosis through various evasion mechanisms, including the overexpression of multidrug resistance transporters, upregulation of inhibitor of apoptosis proteins that inhibit caspase activity, and hyperactivating antiapoptotic signaling pathways such as phosphoinositide 3-kinase/AKT/mammalian target of rapamycin, mitogen-activated protein kinase, and NF-κB [45,46]. This resistance to apoptosis significantly contributes to the failure of current therapies in completely eradicating tumors [16,45]. Therefore, activating alternative cell death pathways is an attractive strategy to overcome apoptosis resistance in CSCs. Necroptosis is a form of controlled necrosis mediated by death receptors [15]. Several recent studies have revealed that necroptosis induction contributes to suppressing therapeutic resistance in CSCs. For example, compounds such as nickel(II)-dithiocarbamate phenanthroline and Os(II)-bathophenanthroline complexes have induced necroptotic cell death in breast CSCs [17,47]. The inhibitor 673A, targeting the ALDH1A enzyme family, has been shown to cause necroptosis in ovarian CSCs by inducing mitochondrial uncoupling proteins and reducing oxidative phosphorylation. Moreover, it has demonstrated synergy with cisplatin chemotherapy [18]. These studies highlight the potential of inducing necroptosis as an effective strategy to eradicate apoptosis-resistant LCSCs. Interestingly, our results showed that low doses of EAHDT induce RIPK3/MLKL-mediated necroptosis, whereas higher doses trigger a transition from necroptosis to caspase-dependent apoptosis in Huh7-derived LCSCs. Previous reports revealed that several anticancer compounds, including apigetrin, gambogenic acid, and shikonin, lead to cancer cell death via the activation of both apoptotic and necroptotic pathways [48,49,50]. In addition, reciprocal backup mechanisms between necroptosis and apoptosis functions under certain conditions have been reported, including energy depletion and changes in therapeutic dose [51]. Thus, EAHDT has the potential to induce both necroptosis and apoptosis of Huh7 LCSCs in a dose-dependent manner. This effect of EAHDT was also demonstrated in Hep3B-derived LCSCs. Cell-death analysis by flow cytometry and H&E staining results showed that low doses of EAHDT induced necroptosis, whereas higher doses caused a transition from necroptosis to apoptosis in Hep3B-derived LCSCs (Supplementary Materials, Figure S2). In the low-dose range, the necroptotic effect of EAHDT may contribute to the efficient elimination of apoptosis-resistant LCSCs. We also evaluated the effect of sorafenib, a multi-kinase inhibitor clinically used for the treatment of patients with advanced HCC, on Hep3B- and Huh7-derived LCSCs. The results showed that sorafenib inhibited the proliferation (IC_50_: 2.9 and 3.3 µM, respectively) and tumorsphere formation of Hep3B- and Huh7-derived LCSCs (Supplementary Materials, Figures S1 and S3). However, sorafenib only caused apoptotic cell death in both LCSCs, indicating that the growth inhibition mechanism of EAHDT in LCSCs is different from that of sorafenib (Supplementary Materials, Figures S2 and S3).

Accumulating evidence has revealed that the activation of the hepatocyte growth factor receptor, c-MET, plays a critical role in the acquisition of stem-like features of cancer cells [37]. c-MET, a receptor tyrosine kinase, activates several downstream signaling pathways that promote the expression of CSC pluripotency markers, including Sox2, Oct4, and Nanog. This activation enhances the self-renewal capacity and malignant characteristics of CSCs [4,52]. Targeting c-MET activity, either with small molecule inhibitors or RNA interference, has been shown to suppress the cancer stem-like phenotype by downregulating CSC markers, including CD133, CD44, ABCG2, Sox2, and ALDH1 [53]. Moreover, c-MET signaling is associated with the growth and chemoresistance of LCSCs [54]. Recent studies reported the potential of ethanol extract from *Diospyros kaki* leaves in reducing stemness markers and increasing the cytotoxic effects of sorafenib by blocking c-MET signaling in HCC [55]. Therefore, inhibiting c-MET-mediated signaling pathways presents a powerful strategy to inhibit the growth and maintenance of LCSCs. In line with this, the results of this study show that EAHDT effectively inactivates c-MET and its critical downstream effectors, including ERK1/2, AKT, and NF-κB, and it also significantly reduces the levels of cancer stemness markers, such as CD133, integrin α6, Sox2, Oct4, Nanog, and ALDH1A1, in Huh7-derived LCSCs. This suggests that EAHDT can inhibit the survival and growth of LCSCs by downregulating stem cell markers through c-MET signaling inactivation.

**Figure 9:**
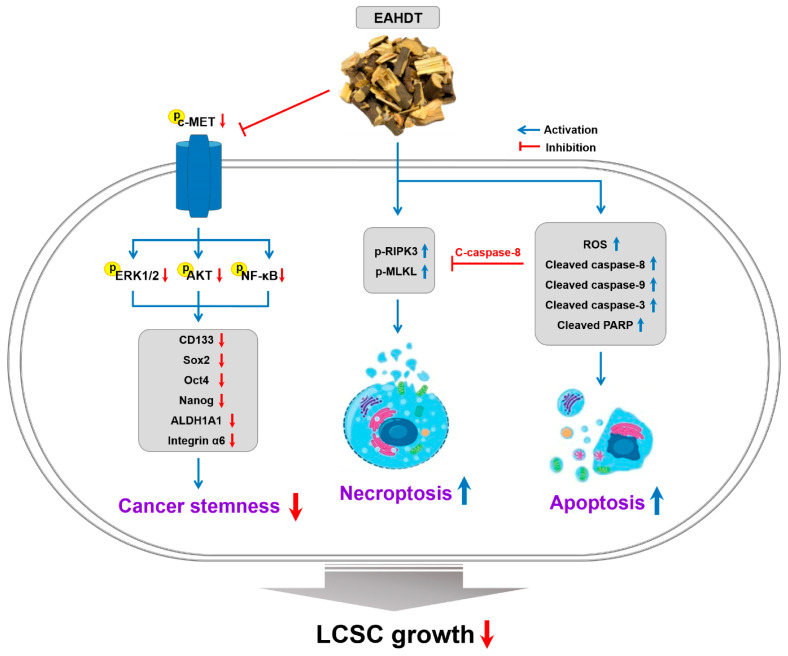
Proposed molecular mechanism for the growth-inhibitory activity of EAHDT against Huh7-derived LCSCs.

In summary, this is the first study to demonstrate the in vitro and in vivo anti-CSC activity of HDT in HCC. The research assessed the inhibitory effects of ethanol, chloroform, and ethyl acetate extracts of HDT branches on the proliferation of Huh7-derived LCSCs. Additionally, we identified the concentration-dependent necroptotic and apoptotic effects of EAHDT in Huh7 LCSCs, elucidating the underlying molecular mechanisms (Figure 9). Additionally, EAHDT was shown to suppress the stem-like characteristics of Huh7 HCC cells through the downregulation of c-MET signaling and stemness markers. Consequently, these research findings suggest that EAHDT holds significant potential as a pharmaceutical resource for the development of natural medicines aimed at preventing and treating HCC by targeting LCSCs. However, considering that EAHDT contains trace amounts of ampelopsin and quercetin, both well-established active ingredients of HDT, it is important to identify the distinct major active constituents responsible for the anti-CSC activity of EAHDT in HCC. This elucidation is necessary for a comprehensive understanding of its mechanism of action. Moreover, in vivo studies using tumor xenograft mouse models are needed to definitively verify the efficacy and safety of EAHDT for the treatment of HCC.

## 5. Conclusions

Our current study identified, for the first time, the growth-inhibitory activity and underlying molecular mechanisms of EAHDT against Huh7-derived LCSCs. EAHDT suppresses the in vitro self-renewal ability of Huh7 LCSCs and reduces in vivo tumor growth in an Huh7 LCSC-transplanted CAM model. EAHDT not only inhibits Huh7 LCSC proliferation by arresting the cell cycle in the G0/G1 phase but it also induces RIPK3/MLKL-mediated necroptosis and caspase-dependent apoptosis of Huh7 LCSCs in a concentration-dependent manner. Furthermore, EAHDT inhibits the expression of CSC markers, including CD133, integrin α6, Sox2, Oct4, Nanog, and ALDH1A1. It also inactivates c-MET and its key downstream effectors, including ERK1/2, AKT, and NF-κB, in Huh7 LCSCs. Based on these results, we propose that EAHDT has the potential to prevent and treat HCC by targeting LCSCs.

## Data Availability

The data that support the findings of this study are available from the corresponding author upon reasonable request.

## Supplementary Materials

The following supporting information can be downloaded at: https://www.mdpi.com/article/10.3390/cells13010022/s1, Figure S1. Effects of EAHDT and sorafenib on proliferation and tumorsphere formation of Hep3B-derived LCSCs. (A,B) Hep3B LCSCs were exposed to different concentrations of (A) EAHDT or (B) sorafenib for 7 days. Cell proliferation was analyzed using the CellTiter-Glo^®^ luminescence assay. The formed tumorspheres were observed and counted. * *p* < 0.05, *** *p* < 0.001 vs. the control; Figure S2. Effects of EAHDT and sorafenib on cell death of Hep3B-derived LCSCs. (A) Hep3B LCSCs were exposed to EAHDT or sorafenib for 48 h. Cell death was analyzed using the Guava^®^ Muse^®^ Cell Analyzer. (B) Hep3B LCSCs were exposed to EAHDT or sorafenib for 24 h. Cell morphology was observed by staining with H&E. Necroptotic and apoptotic cell morphologies are indicated by red and white arrows, respectively. * *p* < 0.05, ** *p* < 0.01, *** *p* < 0.001 vs. the control; Figure S3. Effect of sorafenib on proliferation, tumorsphere formation, and cell death of Huh7-derived LCSCs. (A) Huh7 LCSCs were exposed to different concentrations of sorafenib for 7 days. Cell proliferation was analyzed using the CellTiter-Glo^®^ luminescence assay. The formed tumorspheres were observed and counted. (B) Huh7 LCSCs were exposed to sorafenib for 48 h. Cell death was analyzed using the Guava^®^ Muse^®^ Cell Analyzer. (C) Huh7 LCSCs were exposed to sorafenib for 24 h. Cell morphology was observed by staining with H&E. Apoptotic cell morphology is indicated by white arrow. * *p* < 0.05, ** *p* < 0.01, *** *p* < 0.001 vs. the control.
