# Supplementary material for: Hovenia dulcis Suppresses the Growth of Huh7-Derived Liver Cancer Stem Cells by Inducing Necroptosis and Apoptosis and Blocking c-MET Signaling
Source: Cells. 2023 Dec 21;13(1):22. doi: 10.3390/cells13010022 (PMC10778038; doi:10.3390/cells13010022)
Supplement: Supplementary file 1 [file cells-13-00022-s001.zip › cells-2706909-supplementary.pdf]

Article

Hovenia dulcis Suppresses the Growth of Huh7-Derived Liver Cancer Stem Cells by Inducing Necroptosis and Apoptosis and Blocking c-MET Signaling

Mikyoung Kwon [1] and Hye Jin Jung [1,2,3,*]

1 Department of Life Science and Biochemical Engineering, Graduate School, Sun Moon University, Asan 31460, Republic of Korea; alal5544@sunmoon.ac.kr
2 Department of Pharmaceutical Engineering and Biotechnology, Sun Moon University, Asan 31460, Republic of Korea
3 Genome-Based BioIT Convergence Institute, Sun Moon University, Asan 31460, Republic of Korea
* Correspondence: poka96@sunmoon.ac.kr; Tel.: +82-41-530-2354; Fax: +82-41-530-2939

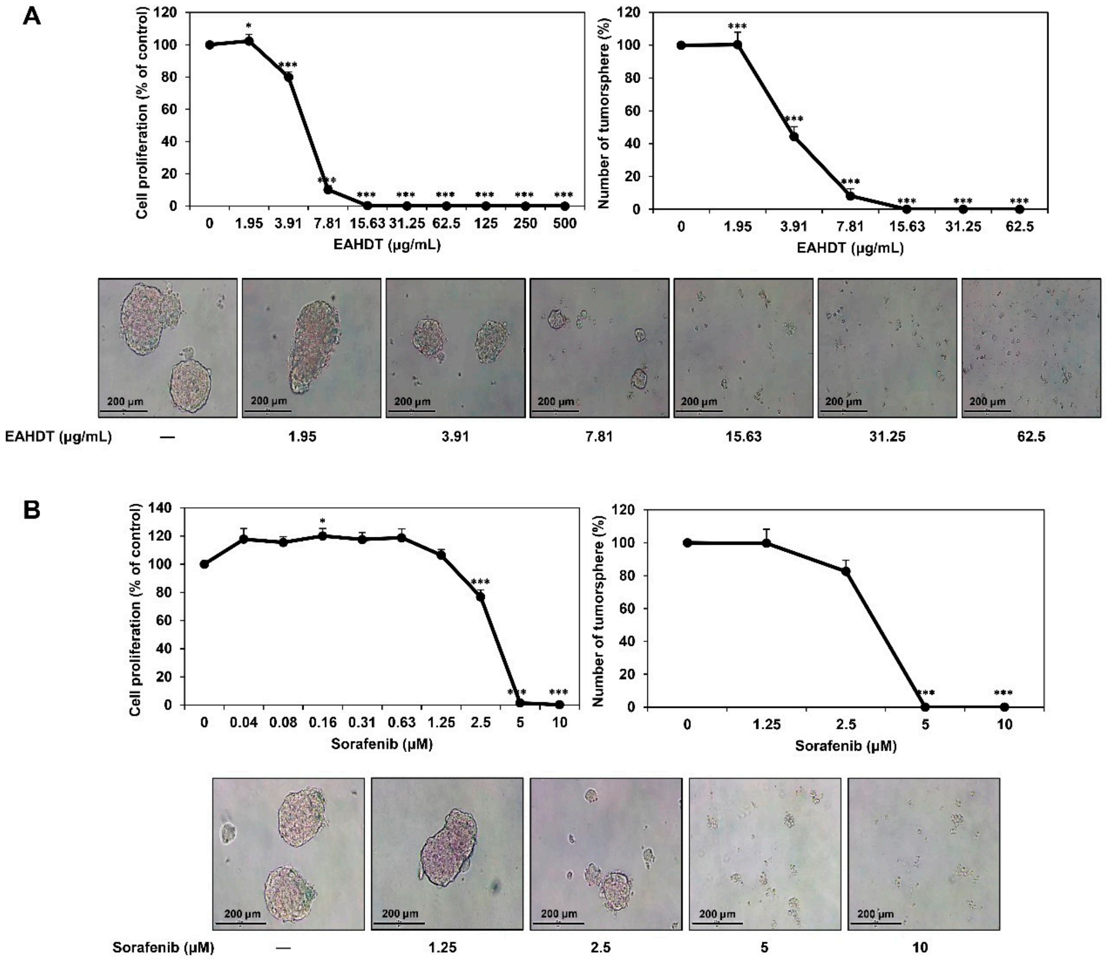

Figure S1. Effects of EAHDT and sorafenib on proliferation and tumorsphere formation of Hep3B-derived LCSCs. (**A,B**) Hep3B LCSCs were exposed to different concentrations of (**A**) EAHDT or (**B**) sorafenib for 7 days. Cell proliferation was analyzed using the CellTiter-Glo® luminescence assay. The formed tumorspheres were observed and counted. * $p < 0.05$, *** $p < 0.001$ vs. the control.

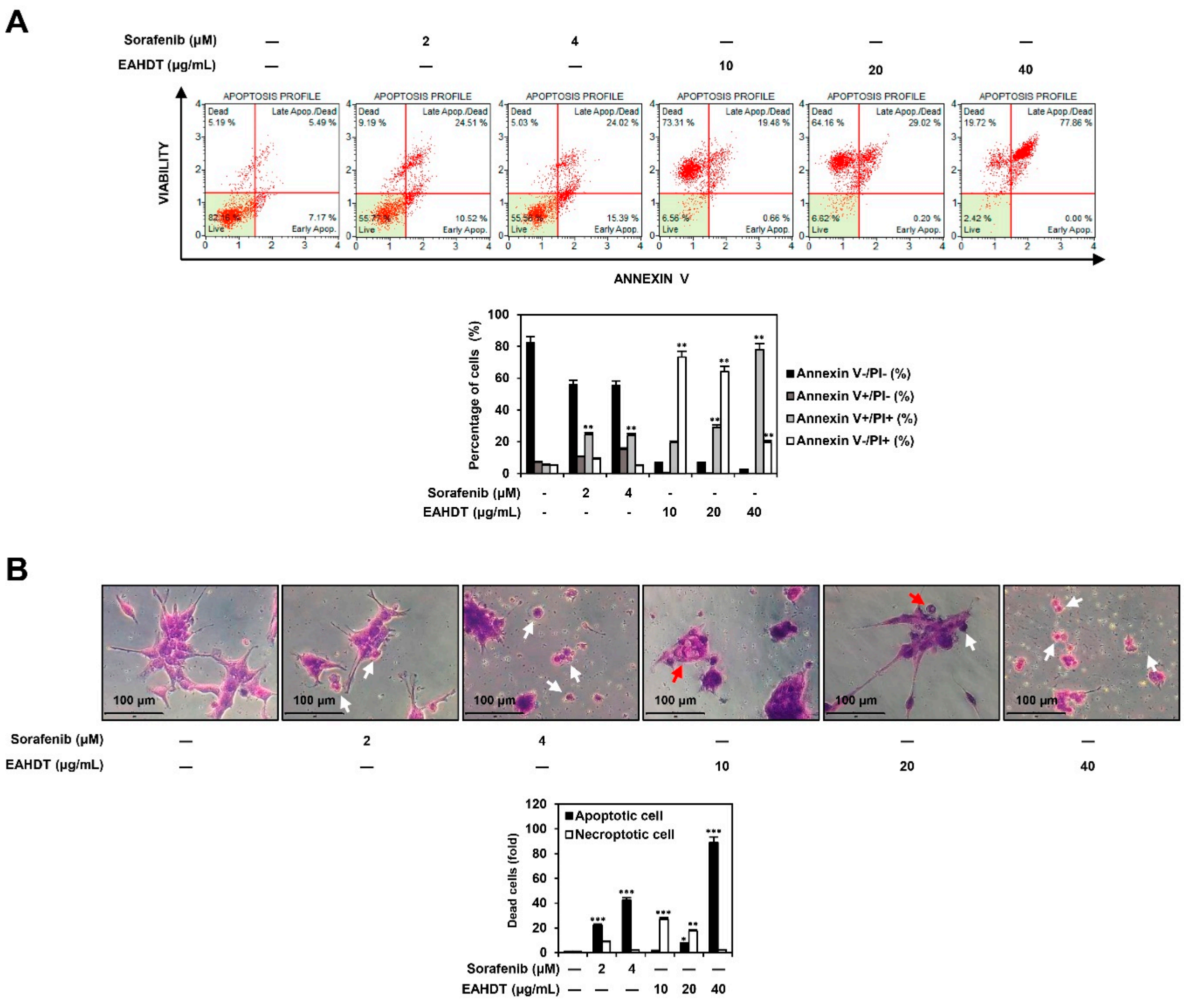

Figure S2. Effects of EAHDT and sorafenib on cell death of Hep3B-derived LCSCs. (**A**) Hep3B LCSCs were exposed to EAHDT or sorafenib for 48 h. Cell death was analyzed using the Guava® Muse® Cell Analyzer. (**B**) Hep3B LCSCs were exposed to EAHDT or sorafenib for 24 h. Cell morphology was observed by staining with H&E. Necroptotic and apoptotic cell morphologies are indicated by red and white arrows, respectively. * $p < 0.05$, ** $p < 0.01$, *** $p < 0.001$ vs. the control.

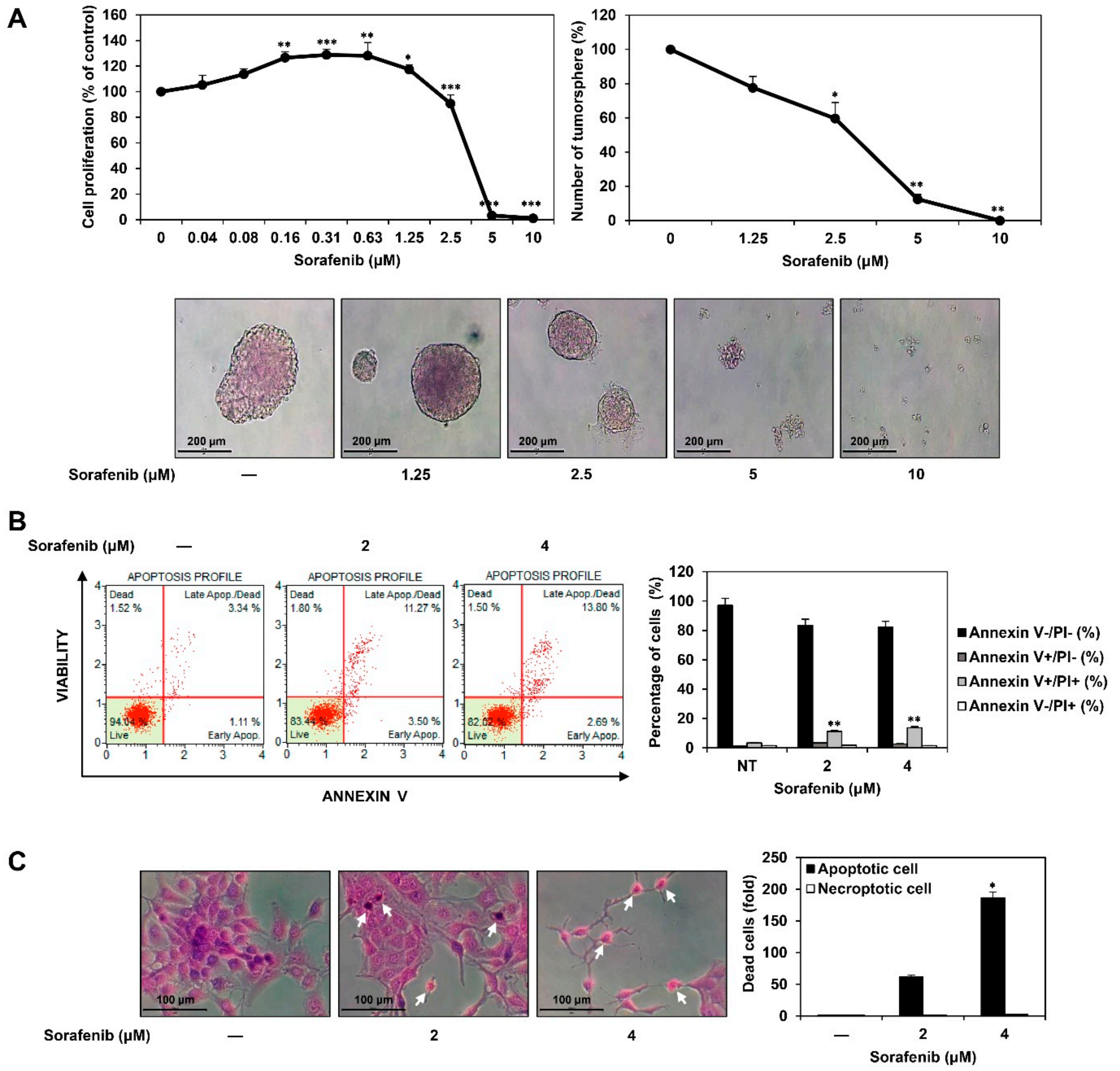

Figure S3. Effect of sorafenib on proliferation, tumorsphere formation, and cell death of Huh7-derived LCSCs. (**A**) Huh7 LCSCs were exposed to different concentrations of sorafenib for 7 days. Cell proliferation was analyzed using the CellTiter-Glo® luminescence assay. The formed tumorspheres were observed and counted. (**B**) Huh7 LCSCs were exposed to sorafenib for 48 h. Cell death was analyzed using the Guava® Muse® Cell Analyzer. (**C**) Huh7 LCSCs were exposed to sorafenib for 24 h. Cell morphology was observed by staining with H&E. Apoptotic cell morphology is indicated by white arrow. * $p < 0.05$, ** $p < 0.01$, *** $p < 0.001$ vs. the control.
